# Diagnostic efficacy of imaging and biopsy methods for peritoneal mesothelioma in a calf

**DOI:** 10.1186/s12917-019-2195-z

**Published:** 2019-12-19

**Authors:** Yasuhiro Morita, Sadamu Sugiyama, Takeshi Tsuka, Yoshiharu Okamoto, Takehito Morita, Yuji Sunden, Takashi Takeuchi

**Affiliations:** 1 Department of Animal Sciences, Graduate School of Bioagricultural Sciences, Nagoya University, Furo-cho, Chikusa-ku, Nagoya, Japan; 2 Asian Satellite Campuses Institute, Nagoya University, Furo-cho, Chikusa-ku, Nagoya, Japan; 3 Maniwa Veterinary Clinic, Okayama Prefectural Federation Agricultural Mutual Aid Association, 794-1 Egawa, Maniwa-City, Okayama Japan; 4 Department of Veterinary Clinical Medicine, School of Veterinary Medicine, Faculty of Agriculture, Tottori University, 4-101, Koyama-Minami, Tottori, Japan

**Keywords:** Calf, Computed tomography, Laparoscopy, Peritoneal mesothelioma, Ultrasonography

## Abstract

**Background:**

Peritoneal mesothelioma is a rare abdominal disease; that occasionally occurs congenitally in younger calves. Cytologic examination of peritoneal effusion (PE) was utilized to diagnose this disease, and was not diagnostic. Diagnostic accuracy has been elevated by recent use of ultrasonography (US), despite most diagnoses have been obtained post-mortem in slaughter houses or during clinical necropsy. In humans, ante-mortem diagnosis is highly associated with clinical use of computed tomography (CT) and laparoscopy together with imaging-assisted biopsy. The present report evaluates the diagnostic applicability of CT and laparoscopy as well as US via the practical application of these imaging modalities in an affected calf, and compares the cytologic and histologic findings among in PE, and specimens obtained from fine-needle aspiration and core-needle biopsy. In addition, the present results were reviewed in comparison with those of previous bovine and human reports.

**Case presentation:**

A 58-day-old male Japanese black calf presented first with scrotal swelling, followed by progressive abdominal distention. Abnormalities of the case included: 1) accumulation of anechoic PE inside the swollen scrotum and abdomen; 2) formation of multiple echogenic nodules within the peritoneal membrane based on US images; 3) presence of hyper-dense spots (suspected calcification) along the margins of the nodules; 4) anatomic connections between intra-abdominal nodular lesions and the swollen tunica vaginalis via the inguinal region based on CT images; 5) serosanguineous-colored and less-turbid characteristics of PE; and 6) formation of multiple nodules over all of the serosa of the rumen as well as the peritoneal wall based on laparoscopic views. Fine-needle aspiration and core-needle biopsy were successfully performed under US and laparoscopic observations, respectively. Histology findings of the core-needle biopsy specimen appeared more indicative (characterization of tubular structures comprised of cubical or columnar abnormal mesothelial cell linings) diagnostically of peritoneal mesothelioma than did findings of the fine-needle aspiration specimen.

**Conclusions:**

To the best of our knowledge, this report is the first description of clinical applications of CT and laparoscopy to diagnose peritoneal mesothelioma in a calf. Laparoscopy enhanced the diagnostic accuracy due to clear gross visualization of the intra-abdominal abnormalities and applicability to imaging-guided core-needle biopsy.

## Background

Stenström first described peritoneal mesothelioma in a 3-day-old calf in 1921 [1]. Mesotheliomas are derived from cells of the serosal linings of the pericardial, pleural, and peritoneal cavities [2]. In bovines, mesotheliomas are classified as the congenital type, affecting calves aged 10 days to 8 months [1, 3–5], and the adult type, affecting bovines beyond the first year of life [2, 6–11]. The majority of mesotheliomas in bovines are the congenital type, occurring within the peritoneum in approximately 94% of affected calves [1].

Accumulation of peritoneal effusion (PE) is a common clinical sign of peritoneal mesothelioma in bovines [1, 2, 4, 7, 9, 11]. Cytologic and biochemical examinations for PE were commonly utilized to diagnose mesothelioma in the past but were not diagnostic in bovine [1, 2, 4, 7, 9, 11] or human patients [12–15]. In most bovine cases, diagnoses are obtained post-mortem in the slaughter-house or during clinical necropsy [1–6, 10, 11]. A variety of imaging techniques have been applied for the diagnosis of mesothelioma, resulting in enhanced diagnostic accuracy in human medicine [12–19]. The imaging modalities of choice in observations of mesothelioma in humans are computed tomography (CT) [12–18] and laparoscopy [15, 17–19]. Recently, clinical applications of ultrasonography (US) to bovine cases have included ante-mortem observations of the formation of variably sized nodules in the serosal or peritoneal membranes, and various amounts of PE [7–9]. However, to our knowledge, CT and laparoscopy have not been applied to the diagnosis of bovine peritoneal mesothelioma. The purpose of the present study, therefore, was to evaluate the clinical applicability of CT and laparoscopy, as well as US, to the ante-mortem diagnosis of peritoneal mesothelioma. In addition, the cytologic or histologic findings of PE, fine-needle aspiration specimen, and core-needle biopsy specimen were compared based on the similarity with necropsy specimen for assessment of the diagnostic efficacy in examination of these tissues collected ante-mortem. These findings were reviewed by comparison with the previous human and bovine reports.

## Case presentation

A 58-day-old male Japanese black calf showed sudden loss of appetite. Body temperature was recorded as 40.8 °C, and both scrotal sacs were enlarged and felt soft upon palpation, initially causing suspicion of orchitis. The elevated body temperature and decreased appetite normalized following daily injections of a non-steroidal anti-inflammatory drug and antibiotic for 3 days. However, the calf showed progressive scrotal enlargement and abdominal distension; the anorexia resumed after the daily injections were discontinued. On day 18, the calf was admitted for imaging examinations.

On day 18, a complete blood count revealed a slight elevation of the red blood cell (RBC) count and thrombocytosis, but normal white blood cell (WBC) count (Table 1) [20, 24]. High level of lactate dehydrogenase (LDH), hypoproteinemia and hypoalbuminemia were evident upon serum biochemical examination [20, 22].

**Table 1:** Blood and ascitic fluid states in this case

	Blood		Ascitic fluid	
	This case	Reference values	This case	Reference values
Red blood cell count (× 10^4^/μl)	1249	853 ± 101 [20]	31	
White blood cell count (/μl)	6300	6500–11,500 [20]	500	670–4900 [21]
Hemoglobin (g/dl)	13.1	9.8 ± 1.3 [20]	0.3	
Hematocrit (%)	41.7	30 ± 5 [20]	0.9	
Platelet count (×10^4^/μl)	107	68.1 ± 25.2 [20]	–	
Urea nitrogen (mg/dl)	24.4	4.2–17.7 [22]	27.3	
Creatinine (mg/dl)	0.85	0.59–1.28 [22]	0.92	
Total protein (g/dl)	4.1	4.7–6.9 [22]	2.0	0.56–4.18 [21]
Albumin (g/dl)	2.6	2.7–3.7 [22]	1.2	0.27–2.39 [21]
Globulin (g/dl)	1.5	1.6–3.6 [22]	0.8	
A/G ratio	1.7	0.78–1.74 [22]	1.5	
Aspartate transaminase (U/l)	137	14.9–103.1 [22]	231	
γ-glutamyltranspeptidase (U/l)	25	0–91.7 [22]	95	
Calcium (mg/dl)	9.5	9.4–12.1 [22]	8.0	
Phosphorus (mg/dl)	9.6	7.0–10.8 [22]	9.5	
Creatine kinase (U/l)	329	29.8–302.5 [22]	39	12.0–167.4 [21]
Lactate dehydrogenase (U/l)	1264	575.8 ± 122.4 [20]	2544	233–960 [21]
SAAG (g/dl)	–		1.4	2.03 [23]

**Fig. 1:**
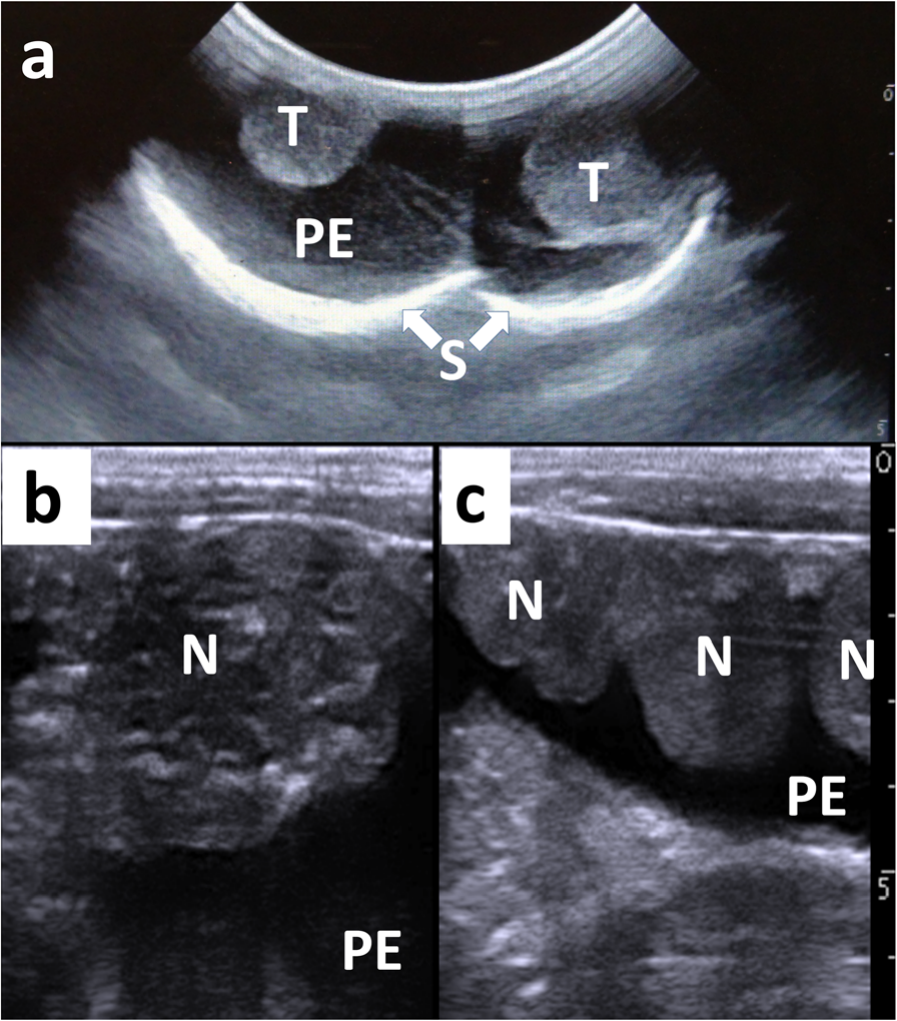
Trans-scrotal ultrasonographic image of the swollen scrotum (**a**), and percutaneous ultrasonographic images of the abdominal cavity (**b** and **c**). **a** Anechoic peritoneal effusion (PE) is seen within the space between the echogenic walls of the scrotum (S) and the testicle (T). **b** Multiple echogenic spots are evident within a 4-cm nodule (N) derived from the echogenic wall of the peritoneum, resulting in slight acoustic shadowing. **c** Multiple 2- to 3-cm nodules (N) are seen in the abdominal cavity, which is filled with an anechoic peritoneal effusion (PE). Scale: 10 mm

A portable-type US device (MyLab™One VET, Esaote Co., Genova, Italy) was applied to the swollen scrotum using a 6.6 MHz convex probe, and the abdomen a using a 6.0 MHz linear probe in a standing position. Transverse trans-scrotal US imaging revealed accumulation of anechoic effusion in the space between the scrotal wall and the testicles, which appeared round in imaging (Fig. 1a). The tunica vaginalis was markedly thickened at the base of the scrotum. Percutaneous US images of the abdominal cavity revealed multiple formations of echogenic nodules derived from the smooth hyperechoic line of the peritoneum. There was slight acoustic shadowing associated with the hyperechoic spots scattered in the nodules (Fig. 1b). The PE appeared anechoic on imaging, with a small amount of echogenic debris, and filled the abdominal cavity. Within the caudal abdominal cavity (near the pelvic cavity), multiple small to large nodules were aligned in the anechoic PE (Fig. 1c). Under US imaging, PE was collected by percutaneous abdominocentesis with an 18-gauge needle. Subsequently, the nodular tissue was obtained by fine-needle aspiration with a 22-gauge needle (Terumo spinal needle, Terumo Co., Tokyo, Japan).

**Fig. 2:**
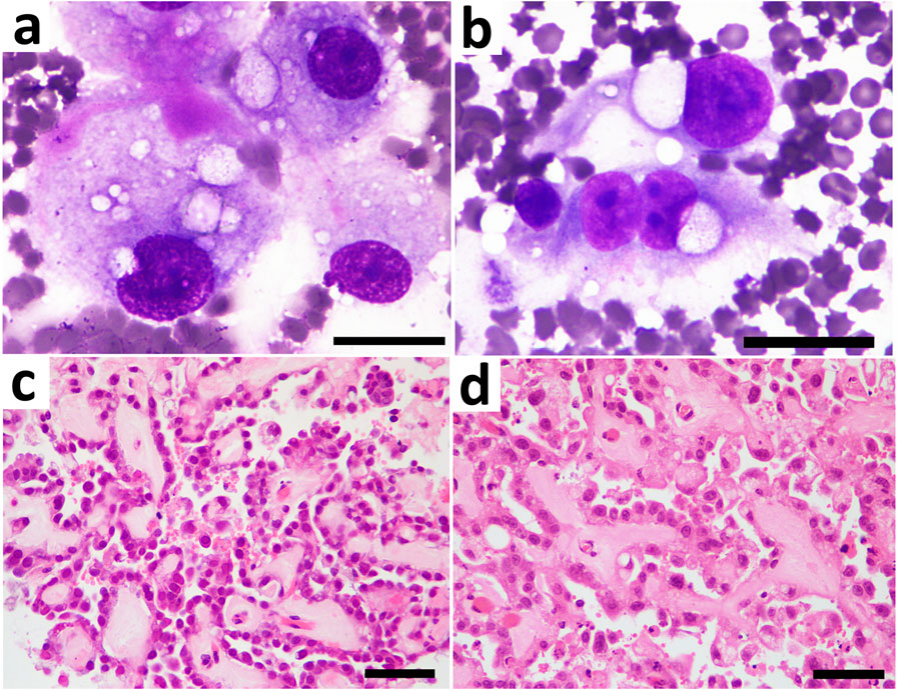
Cytology of the peritoneal effusion (**a**); specimen obtained from ultrasound-guided fine-needle aspiration (**b**); histopathology of the specimen obtained from laparoscopy-guided Tru-cut biopsy (**c**), and that of peritoneal nodules collected at necropsy (**d**). **a** Small focus of large epithelioid cells is seen, with background comprised of large numbers of erythrocytes (Giemsa). The cells are large and have a round to oval-shaped nucleus. **b** Large epithelioid cells are seen together with large numbers of erythrocyte (Giemsa). The cells have a large round to oval-shaped nucleus. The cytoplasm is clear and/or has some vacuoles. **c** Cubical or columnar cells, which resemble mesothelial cells, align and form papillary structures (HE). **d** There are epithelial linings of cubical or columnar mesothelial-like cells with some nuclear debris and pale eosinophilic stroma (HE). Bars = 250 μm (**a**, **b**) and 50 μm (**c**, **d**), respectively

The PE was clear and serosanguineous. The PE WBC level was low, and the LDH level was high (Table 1) [21]. The PE levels of total protein and albumin were normal, but the serum-ascites albumin gradient (SAAG), which is calculated by subtraction of the PE albumin level from that of the serum, was low [21, 23]. The sediment of the PE was applied onto glass slides and stained with Giemsa stain. Small aggregations of large, square to round, epithelioid cells were observed between the RBCs and neutrophils. These cells had abundant cytoplasm with vacuoles and prominent nucleoli (Fig. 2a). The border of the cells was unclear, and occasional microvilli-like structures were observed on the surface. Similar findings were also seen in fine-needle aspiration samples collected under the guide of US (Fig. 2b). However, larger cell aggregates and fewer neutrophils were observed in the fine-needle aspiration specimens compared with the PE specimens.

**Fig. 3:**
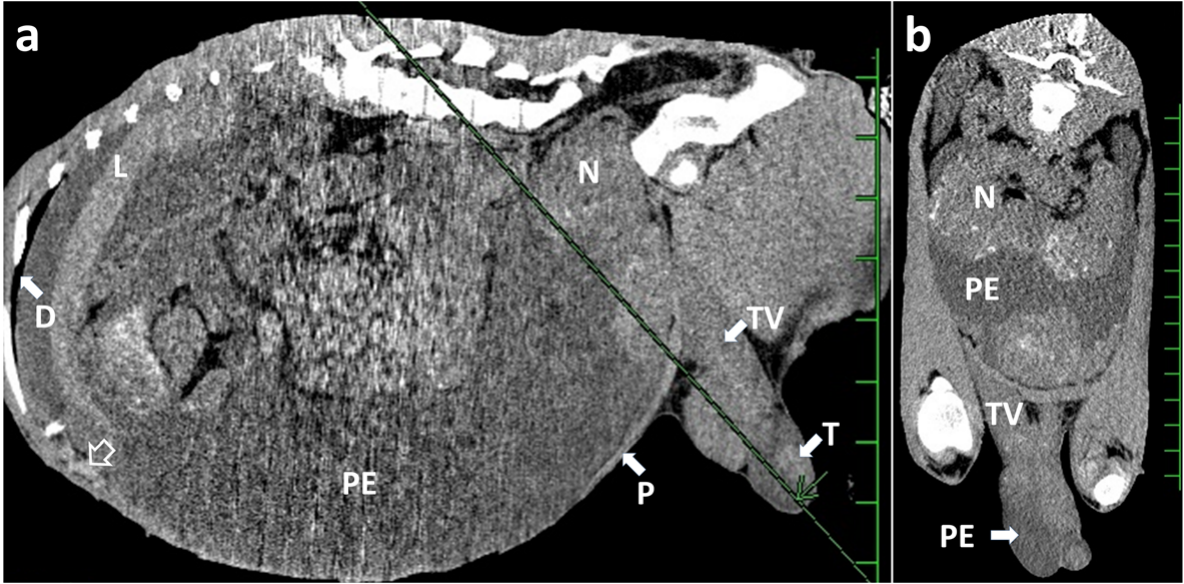
Sagittal computed tomography (CT) visualizing the abdominal cavity (**a**), and transverse CT obtained from a line established along the tunica vaginalis (TV) (**b**). **a** Peritoneal effusion (PE) is accumulated throughout the abdominal cavity. Enlarged nodules (N) are seen densely around the caudal region of the abdominal cavity and connected with the swollen tunica vaginalis (TV) via the inguinal region. Irregular and thickened walls (empty arrow) are seen within the cranial region of the peritoneum (P) and the diaphragm (D). L: liver. T: testicle. Scale: 50 mm. **b** Multiple small hyperdense spots are evident along the margins of the dorsal enlarged nodules (N). Peritoneal effusion (PE) is seen in the abdominal cavity and the scrotum. The tunica vaginalis (TV) is swollen. Scale: 25 mm

The animal was placed in a right recumbent position under general anesthesia with 2–3% isoflurane via an endotracheal tube, inserted after sedation with an intravenous injection of xylazine hydrochloride (0.2 mg/kg). CT examination using a helical scanner (Pronto SE, Hitachi Co. Ltd., Tokyo, Japan) revealed accumulation of PE throughout the abdominal cavity. Dense assemblages of enlarged nodules were predominantly seen in the caudal abdominal cavity and connected to the swollen tunica vaginalis via the inguinal region on sagittal images (Fig. 3a). The wall of the border between the ventral diaphragm and the cranial peritoneum appeared irregular and thickened on imaging. Transverse CT images obtained from a line established along the tunica vaginalis showed multiple small hyperdense spots formed along the margins of the nodules (Fig. 3b). Within the swollen scrotum, PE into the apex was observed, and the tunica vaginalis was markedly thickened.

**Fig. 4:**
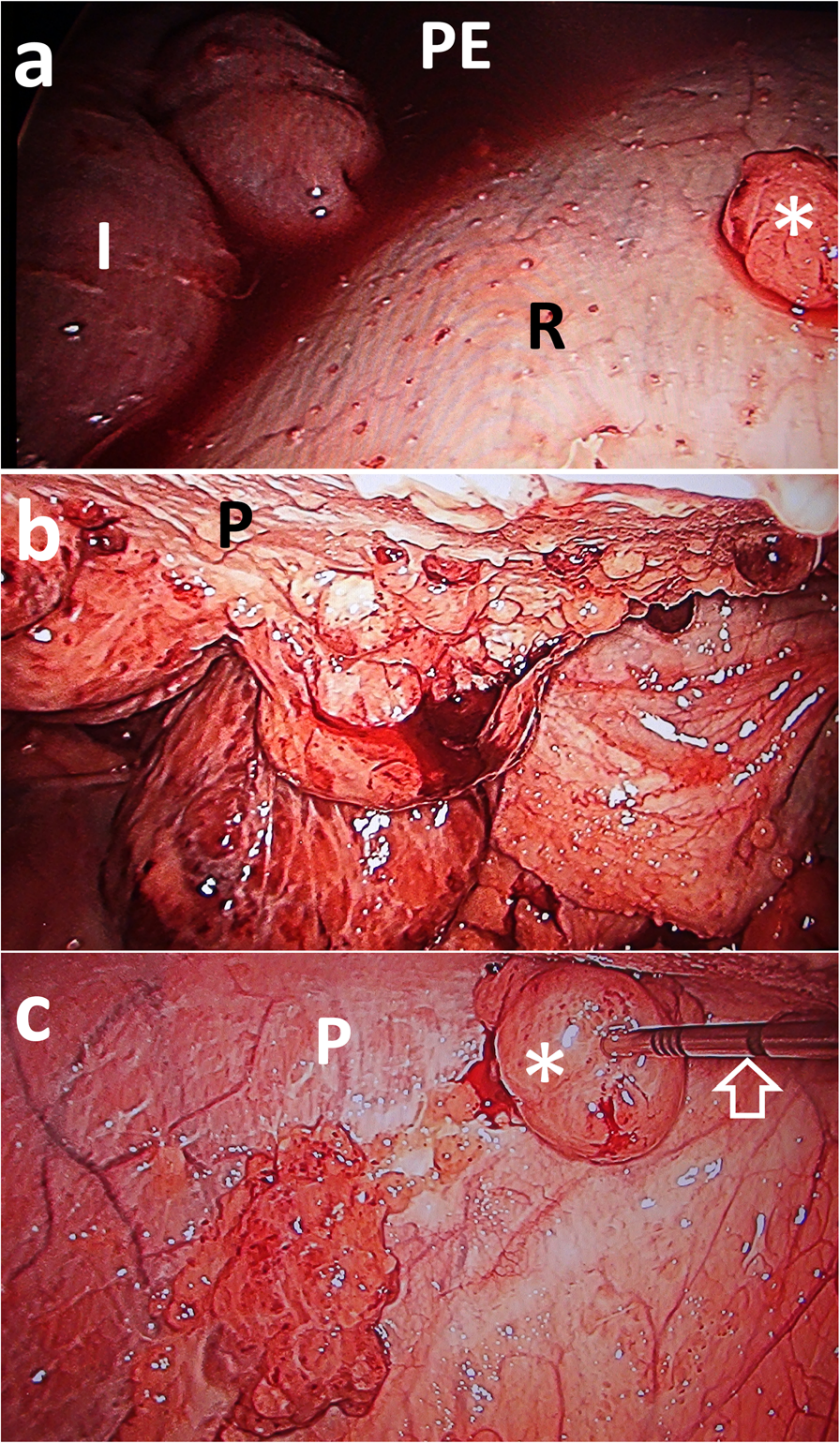
Laparoscopic views of the lumen of the abdomen visualizing the rumen in the cranial cavity (**a**), the caudal cavity (**b**), and Tru-cut biopsy of a nodule (**c**). **a** Nodule (asterisks) is seen throughout the serosa of the rumen (R). Serosanguineous peritoneal effusion (PE) is seen in the space between the rumen and the intestine (I). **b** Multiple small and enlarged nodules are evident throughout the peritoneum (P). **c** Tru-cut biopsy needle (empty arrow) is inserted into a nodule (asterisk) formed in the peritoneum (P) under laparoscopic view

Laparoscopy (1288 HD Camera and L9000 LED Light Source, Stryker Co., Michigan, USA) was performed with the animal in a supine position soon after CT examination (under the same anesthesia). After removal of approximately 20 l of PE via catheter in order to ensure a wider laparoscopic field of view, a 10 mm trocar was introduced approximately 10 cm from the lower right part of the umbilicus. Laparoscopic views of the cranial abdominal cavity revealed multiple nodules throughout the serosa of the rumen (Fig. 4a). The nodules were round shaped with irregular surface and pink or dark red in color. The accumulation of a large amount of serosanguineous PE prevented complete visualization of the cranial region of the abdominal organs (abomasum, liver, and spleen) and the peritoneal walls. Within the caudal abdominal cavity, multiple small to large nodules were evident on the peritoneal walls (Fig. 4b). The peritoneum, in which multiple nodules were present, was characterized by normal, smooth, and pink colored walls on the ventral surface of the abdomen; and abnormal, irregular, and dark red colored walls on the dorsal surface of the abdomen. The nodules were round shaped and smooth, and colored white, pink, and dark red. Small blood vessels were observed on the surfaces of some nodules. Laparoscopy-guided core-needle biopsy was performed on an approximately 2 cm nodule formed on the ventral surface of the abdomen using a Tru-cut biopsy needle (Tru-Core™ II Automatic Biopsy Instrument, Argon Medical Devices Inc., Texas, USA) that was percutaneously inserted (Fig. 4c).

**Fig. 5:**
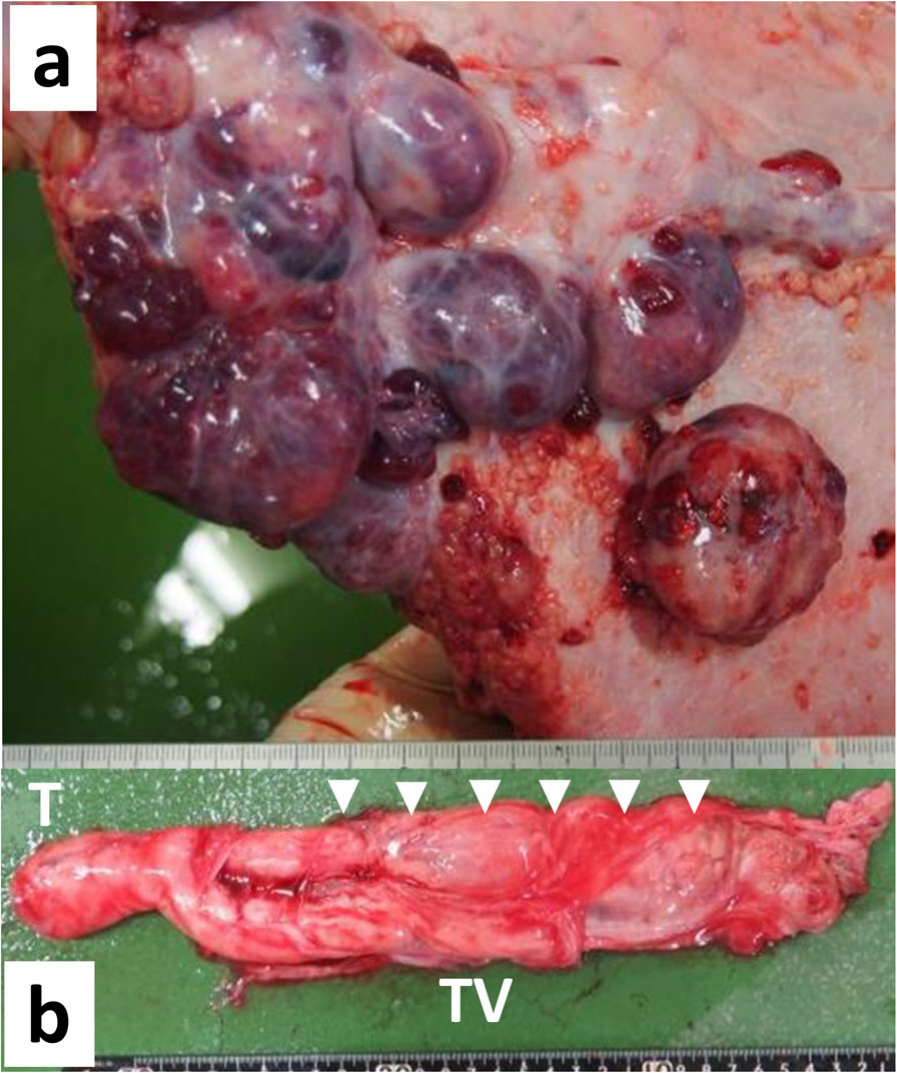
Necropsy appearance of the caudal abdominal cavity (**a**) and the right tunica vaginalis and testicle (**b**). **a** Multiple parenchymal or cystic nodules are evident on the peritoneal walls. **b** The white-colored soft structure (arrowheads) is present along the tunica vaginalis (TV). The testicle (T) is slightly swollen

The animal was died suddenly 1 day after these examinations. Necropsy revealed that numerous (more than 100) nodular lesions were found on the peritoneum with bloody PE in the abdominal cavity (Fig. 5a). The nodules were whitish or reddish in color, and varied in size (maximum 5 × 4 × 4 cm). The nodules were coalescing and disseminating on the serosa and peritoneum. The cut surfaces of the nodules were solid and irregularly lobulated by thin fibrous tissues, and often showed hemorrhage and necrosis. These nodules were also found on the omentum, mesentery, and the surface of several organs, including the liver, rumen, and spleen; however, the borders between the masses and each organ were clear. Both scrotal sacs were enlarged by the infiltration of well-defined similar structures extending from the funiculus spermaticus to the testis (Fig. 5b). Focal, and mild broncho-pneumonia was present in the right lung lobes. The lymph nodes in both thoracic and abdominal cavities were grossly normal.

The pieces of tissues obtained from the laparoscopy-guided core-needle biopsy and the masses collected at necropsy were fixed in formalin and routinely processed for paraffin embedding, then stained by hematoxylin and eosin (HE) stain. Microscopic examination revealed the proliferation of cubical or columnar neoplastic cells in the nodule. These cells were aligned and formed papillary structures (Fig. 2c,d). Furthermore, a few fibrovascular interstitial tissues and mucinous material were seen together with multi-layered neoplastic cells. The cells were morphologically similar to mesothelial cells. They showed strong immunoreaction for cytokeratin (AE1/AE3) and weak immunoreaction for vimentin (data not shown). The neoplastic cells often showed necrosis with debris from the nuclei. Dystrophic calcification was also scattered within the masses. The testes appeared slightly atrophic from the invasion of neoplastic cells into the scrotum, a continuation from peritoneum. Invasion of neoplastic cells into the parenchymal of the testes was not observed. Proliferative lesions were not evident in other abdominal organs including the intestine, pancreas, and genitalia. Finally, histology of the necropsy specimen in the present case revealed that a mucinous background within the nodular lesion was one of the characteristics of mesothelioma. Marked atypia of neoplastic cells with frequent mitosis, as well as multiple and disseminated lesions in the peritoneum support malignancy of the lesion. Based on these gross and histopathlogic findings, the lesions in the present case were diagnosed as malignant mesothelioma.

## Discussion and conclusions

Malignant mesotheliomas generally have a poor prognosis in bovine cases affecting at any anatomical place, because of the extensive invasion, and poor response to various treatments, with the exception of previous therapeutic success using unilateral orchiectomy for two bulls with localized involvements [9]. Thus, the appropriate action for the affected bovine cases must be culling based on the early diagnosis. Peritoneal mesotheliomas may be relatively easily detected in the field as the most common clinical sign is swelling of the abdomen [6–8]. The pathogenesis of abdominal distension due to peritoneal mesotheliomas included both multiple formations of large nodular lesions and accumulations of large amounts of PE [1, 4, 6, 9]. Conversely, small to normal amounts of PE can lead to delayed discovery of early disease, and uncertain determination of onset period, although data regarding the production rate of fluid are lacking in bovines. Imaging diagnosis can contribute to increased diagnostic accuracy in affected cattle exhibiting variety of clinical signs such as abdominal distention and PE.

US was used as an imaging modality for the ante-mortem diagnosis of mesothelioma in cattle [7–9]. Previous uses of US enabled visualization of the thickened and edematous changes in the omentum with fluid-filled cystic structures, as well as the typical pattern such as peritoneal nodularity (commonly multiple nodules) and accumulation of PE [7–9]. In the present case, a large amount of PE might prevent visualization of lesions that can be present within the serosa of the abdominal organs. Specific US pattern of peritoneal nodularity in the present case included multiple echogenic spots in lining of the nodules, indicating calcification. This finding may be one of the diagnostic US characteristics distinguishable from other abdominal diseases; a retrospective study of bovine cases revealed calcification within the nodules in 4 of 15 heads (26.7%) [1]. PEs were commonly anechoic or hypoechoic, suggesting production of transudates associated with peritoneal mesotheliomas, although this was not specific in the bovine cases [7, 8]. US diagnosis of either transudates or exudates based on echogenicity of PEs can be supported by cytologic and biochemical examinations of the effusions [1, 2, 4, 7, 9, 11]. The PE in the present case was determined to be a transudate based on the lack of turbidity [1, 9, 11], WBC count less than 5000/μl [25], and SAAG greater than 1.1 g/dl [26]. In addition, our results support the diagnostic efficacy of trans-scrotal US imaging for male bovine case, visualizing the thickened tunica vaginalis, intra-scrotal accumulation of PE, and intra-scrotal invasion of abdominal masses along the tunica vaginalis [1, 9].

CT is commonly used in humans for the diagnosis of peritoneal mesothelioma because of the characteristic CT findings and usefulness of contrast media [12–15, 17], although CT cannot always provide diagnostic findings in humans [16]. The use of CT for the present case enabled clear visualization of nodular lesions in the pelvic region, the predominantly affected region in previous human and bovine cases [6, 12]. However, CT did not show multiple nodules in the omentum and serosa of the abdominal organs located in the cranial and middle abdomen. “Omental cake” is a specific CT finding indicative of omental infiltration [14, 15]. Our results indicate the difficulty in CT visualization of the serosal lesions due to poor image quality associated with the respiratory motion of the examined animal.

CT is utilized to evaluate prognosis in humans based on the invasion of lesions into the intra-parenchymal layers of organs and extension of lesions throughout multiple body cavities via the involved lymph nodes and ducts [13, 18]. The use of whole-body CT in the affected cattle may allow visualization of distant metastases, characterized as involvement of the thoracic cavity (such as pleural and pericardial membranes or the lungs) and the abdomen, and infiltration into the lymph nodes, although it was not commonly noted in previous cases [1, 2, 8, 10, 11]. In the present case, CT images were suspicious for calcification due to hyperdense spots along margins of the nodules. Foci of calcification are a typical histologic finding in peritoneal mesothelioma [6].

Laparoscopy is applied in approximately half of human patients with peritoneal mesothelioma, providing high diagnostic accuracy [15, 17–19]. Bovine cases have previously been examined by laparotomy, but not by laparoscopy [6, 9]. The previous uses of laparotomy contributed to intra-abdominal observation and sampling for histological examination, but did not allow surgical resection of the abdominal masses as they were too widely distributed for complete resection [6, 9]. Thus, laparoscopy may be more suitable compared to laparotomy for bovine practice, as the main purpose is diagnosis of peritoneal lesions rather than treatment. On laparoscopic observation, the color and turbidity of PEs are utilized to differentiate transudates and exudates, and the degree of lesion distribution in the peritoneal and serosal membranes is useful for both diagnosis and determination of the prognosis of peritoneal mesothelioma [15, 17–19]. In laparoscopic analysis of bovine cases, differential diagnosis between peritoneal mesotheliomas and abdominal lymphomas is necessary, as these two lesions are characterized by multiple nodules within the abdominal organs [4]. In addition, laparoscopic observations of the nodules should be conducted carefully to prevent misdiagnosis of fibrinous peritonitis, chronic granulomatous peritonitis, peritoneal tuberculosis, or other metastatic tumors of serosal membranes due to the similarity of the lesions [2, 27].

Laparoscopy was applied to abomasopexy around 2000 and to biopsy involving the abdomen around 2010 in bovine medicine [28, 29]. These techniques can also be applied in standing cattle with the use of sedation and local anesthesia, although our technique was performed on a recumbent animal under deep anesthesia [28, 29]. In addition, our technique, in which the biopsy needle was percutaneously inserted into the abdomen through a part different from the trocar, is not a simple method for intra-abdominal introduction of a biopsy needle. Centesis associated with needle and port sites can facilitate tumor dissemination [14]. This can be minimized using a one-port assisted core-needle biopsy technique, in which a biopsy forceps is introduced into the channel of the operating endoscope [29].

In the cytological examinations, ultrasound-guided fine-needle aspiration might allow collection of a larger amount of abnormal tissue compared to a fragment exfoliated into PE. However, the cytology of these two specimens was of minimal value for the definitive diagnosis of peritoneal mesothelioma, as expected. PE cytology can provide evidence of atypia and hyperchromasia of mesothelial cells, consistent with reactive mesothelial cell proliferation, but this is non-diagnostic [12, 19]; it has not been successful in the diagnoses of approximately half of human patients [18]. In addition, the cytologic findings of fine-needle aspiration primarily contribute to non-specific diagnoses and frequently include reactive mesothelial cells [18].

In cattle, desquamated mesothelial cells are normally seen in PE, and frequently cannot be differentiated from abnormal mesothelial cells [27]. The cellular component of PEs due to peritoneal mesotheliomas typically included phagocytically active mesothelial cells [9] and normal mesothelial cells [5]. In some bovine cases, abnormal, large cells with round nuclei containing multiple nucleoli are seen in PE, but whether such findings are sufficient evidence for ante-mortem diagnosis is unclear [9]. The difficulty of diagnosis based on cytologic examination of PEs is also evidenced by a previous report describing misdiagnosis of non-septic and active peritonitis [11].

Histology of the necropsy specimen in the present case could contribute to a definitive diagnosis of peritoneal mesothelioma together with immunohistochemistory, in which the neoplastic cells were positive for both epithelial and mesenchymal marker intermediate filaments (cytokeratin and vimentin) [11, 30] In bovines, potential difficulties associated with histologic examinations have been described; variations of mitotic figures were observed rarely [2, 5, 10, 11] or commonly [4], and hyperplastic reactions of mesothelial cells induced by the effects of various abdominal diseases frequently resemble histologic characteristics of peritoneal mesothelioma [27]. However, these difficulties can be minimized by collecting specimens in which the structures are definitively maintained and similar to those formed within the nodules. Based on the present results, core-needle biopsy using a Tru-cut biopsy needle provides higher diagnostic accuracy than can be achieved with specimens from PE and fine-needle aspiration due to the microscopic nature of the examination. Laparoscopy should be utilized to support core-needle biopsy in bovine cases, as it is the most useful imaging technique applicable to core-needle biopsy in human medicine [19]. Ante-mortem diagnosis will be meaningful for reduced economic loss due to prolonged breeding and unsuccessful treatments when imaging and biopsy techniques are conjunctively used in bovine medicine.

## Data Availability

The datasets used and/or analyzed during the current study available from the corresponding author on reasonable request.

## Abbreviations

CT: Computed tomography
LDH: Lactate dehydrogenase
PE: Peritoneal effusion
RBC: Red blood cell
US: Ultrasonography
WBC: White blood cell
